# Double Cervical Adjacent Hydrated Nucleus Pulposus Extrusion (HNPE) in a Yorkshire Terrier

**DOI:** 10.3390/ani14192889

**Published:** 2024-10-08

**Authors:** Domenico Fugazzotto, Marco Tabbì, Pilar Lorena Lozano, Giuseppe Barillaro, Francesco Macrì, Simone Minato

**Affiliations:** 1 Department of Veterinary Sciences, University of Messina, 98168 Messina, Italy; domenicofugazzotto@gmail.com (D.F.); francesco.macri@unime.it (F.M.); 2 Ospedale Veterinario San Francesco, 31038 Paese, Italy; pilarlorenalozano@gmail.com (P.L.L.); minatosimone81@gmail.com (S.M.); 3 Clinica Veterinaria San Giorgio (CVSG), 89121 Reggio Calabria, Italy; info@cvsg.it

**Keywords:** dog, tetraparesis, MRI, canine, HNPE

## Abstract

**Simple Summary:**

Hydrated nucleus pulposus extrusion (HNPE) is a well-known pathological condition in veterinary neurology. Any breed of middle-aged or older dogs (median 9 years old) can be affected; it has an acute spontaneous onset, is rarely exercise-associated, and commonly affects the cervical region, causing tetraparesis or tetraplegia and mild cervical hyperesthesia. This study presents the first documented case of double cervical adjacent HNPEs in a small breed dog. A 9-year-old 6 Kg intact male Yorkshire terrier was referred for an acute onset of non-ambulatory tetraparesis. MR examination of the cervical spine showed two concurrent HNPEs at sites C3-C4 and C4-C5. The dog had a remission of symptoms with conservative therapy and physiotherapy.

**Abstract:**

A 9-year-old Yorkshire terrier was brought to the emergency department for inability to maintain the correct station with acute onset. Neurological examination showed a non-ambulatory tetraparesis, spontaneous proprioceptive deficit in all limbs, and decreased flexor reflex in the forelimbs. The neurological symptoms suggested a cranial cervical spinal cord with suspicion of spinal shock. The clinical differential diagnoses included degenerative (intervertebral disc extrusion), vascular, inflammatory, or neoplastic disease. No pathological findings were evident in the hematobiochemical tests or in the radiograph examination. MRI examination of the cervical spine showed the presence of two adjacent hydrated nucleus pulposus extrusions at C3-C4 and C4-C5 tracts. Treatment included analgesic and non-steroidal anti-inflammatory therapy; movement restriction was initially necessary, followed by physiotherapy. Follow-up at 4 weeks showed complete recovery. A telephone follow-up after 3 months with the owner confirmed the absence of symptoms. This article reports the first double cervical HNPE case in a dog, adding the possibility that the disease may present in this form and the success of conservative treatment as described in the literature.

## 1. Introduction

Intervertebral disk herniation represents one of the most common neurological diseases in dogs, which can be either compressive or non-compressive, caused by the extrusion of a degenerate or nondegenerate nucleus pulposus [1,2]. Among the nondegenerate compressive nucleus pulposus extrusions, hydrated nucleus pulposus extrusion (HNPE) has been described in recent literature [1,3,4,5,6,7,8,9,10,11]. Also known in the past as canine intraspinal discal cist [12], it has been shown that the extruded material consists of gelatinous, well-hydrated nucleus pulposus, which causes varying degrees of spinal cord compression [5]. Any breed of middle-aged or older dogs (median 9 years old) can be affected; it has an acute spontaneous onset, is rarely exercise-associated, and commonly affects the cervical region, causing tetraparesis or tetraplegia and mild cervical hyperesthesia [1,2,6,9]. MRI criteria for diagnosis have been well-established [1,2,5,8,13,14] with the presence of extradural material located ventral and midline to the spinal cord, dorsally to the intervertebral disk, typically hyperintense in T2-weighted sequences and isointense to the nucleus pulposus in all the other sequences. When present, almost pathognomonic is the bilobed seagull appearance in transverse sequences; narrowing of the intervertebral space and reduction of the volume of the nucleus pulposus can be associated too [13]. Treatment can be surgical or conservative, and nowadays, there are no guidelines for which one should be used in which circumstances; in two studies [7,10], independently of the neurological status, there was no difference in outcome for dogs treated conservatively and surgically. Surgical treatment is often chosen when there is evidence of severe spinal cord compression associated with severe neurological signs and respiratory compromise [3,15]. The outcome is generally good, with a median time of recovery of independent ambulation in 6 days [7]. An unfavorable prognosis is associated with respiratory compromise. The aim of this case report is to describe the clinical features, diagnosis, treatment, and outcome of a non-ambulatory dog presenting with two simultaneous, adjacent cervical HNPEs.

## 2. Case Description

**Figure 1:**
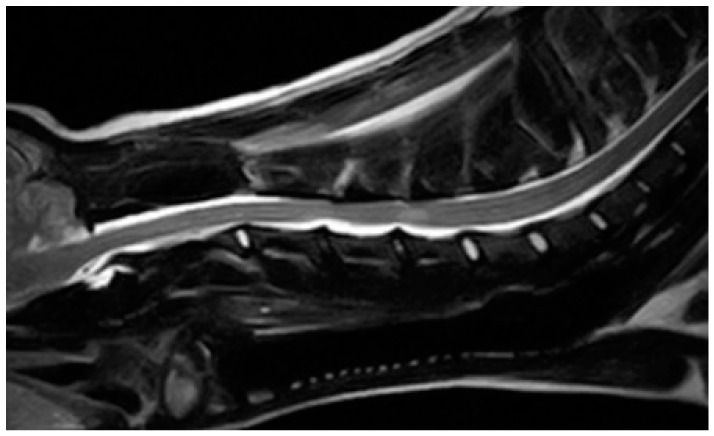
T2W sagittal MRI image: Intervertebral disc degeneration at sites C3-C4 and C4-C5. At the level of these sites, ventral extradural HNPEs, which are homogenously hyperintense, lie immediately dorsal to the affected intervertebral discs and subsequent obliteration of the dorsal subarachnoid spaces. Presence of intraparenchymal lesion at the level of C4-C5 inter-somatic space.

A 9-year-old 6 kg intact male Yorkshire terrier was referred for an acute onset of inability to maintain station. Traumatic events were excluded from medical history. Physical examination revealed a good body condition score (3/5). Neurological examination revealed a non-ambulatory tetraparesis, spontaneous proprioceptive deficit in all limbs, and decreased flexor reflex in the forelimbs. The cutaneous trunci reflex was normal. Examinations of the cranial nerves and menace responses were normal. Mild hyperesthesia was present on palpation of the neck. The neurological symptoms suggested a cranial cervical spinal cord lesion between the first cervical (C1) and the fifth cervical (C5) spinal cord segments and suspicion of spinal shock. The clinical differential diagnoses included degenerative (intervertebral disc extrusion), vascular, inflammatory, or neoplastic disease. Complete blood count (Mindray BC-5000, Mindray Medical Italy, Milan, Italy) and serum biochemistry (BT300 Plus, Biotecnica instruments, Rome, Italy) analysis were normal. No alterations were present in radiographs of the cervical spine (MAXIVET DR with FLAT-PANEL SYSTEM^®^, Multimage Srl, Varese, Italy). The patient was sedated with a protocol involving 2 µg/kg i.m. dexmedetomidine (Dextroquillan, 0.5 mg/mL, Fatro S.p.A., Bologna, Italy) and 0.2 mg/kg Midazolam (Midazolam Bioindustria L.I.M., Bioindustria L.I.M., Novi Ligure, Italy). General anesthesia was induced with propofol (Proposure, Boehringer Ingelheim Animal Health Italia s.p.a., Milano, Italy) to achieve orotracheal intubation and maintained with isoflurane (IsoFlo, Zoetis Italia s.r.l., 20124, Milano, Italy) in oxygen with mechanical ventilation. Capnography and halogenates were monitored. The dog was monitored with continuous ECG, pulse oximetry, and non-invasive blood pressure control. MR examination of the cervical spine was performed using a 0.4T (Hitachi Aperto Lucent, Fujifilm Italia Spa, Milan, Italy). The protocol included T2-weighted series (T2W; TR 6000 ms; TE 120 ms) in the sagittal and transverse planes and T1-weighted series (T1W; TR 520 ms; TE 20 ms) in two orthogonal planes before and after intravenous administration of paramagnetic contrast 0.1 mL/kg (Gadovist 1 mmol/mL, Bayer Spa, Milan, Italy). MRI showed intervertebral disc degeneration at sites C3-C4 and C4-C5. At the level of these sites, there was evidence of disc extrusion of hydrated nucleus pulposus, determining significant spinal cord compression (Figure 1).

**Figure 2:**
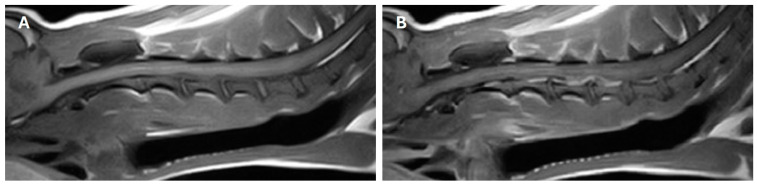
(**A**) T1W sagittal MRI image before paramagnetic contrast: C3-C4 and C4-C5 ventral HNPEs of hypointense signal and (**B**) in T1W sagittal image after paramagnetic contrast show good enhancement, most evident at the C4-C5 site.

The extruded disc material showed a strongly homogeneous hyperintense signal in T2-weighted sequences and hypointense in T1-weighted sequences, with good contrast enhancement. At the C3-C4 site, the spinal cord compression was median, while at the C4-C5 site, it was the right paramedian (Figure 2).

**Figure 3:**
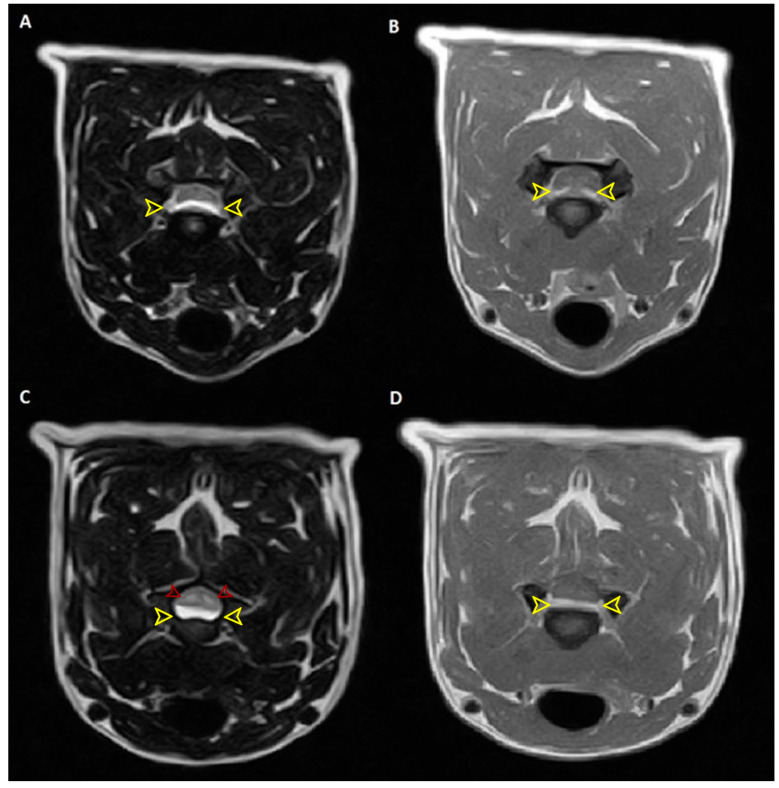
(**A**) T2W transverse image: C3-C4 median spinal cord compression by extruded disc material of hyperintense signal (yellow arrowheads). (**B**) captant enhancement in T1W post-contrast transverse image (yellow arrowheads). (**C**) T2W transverse image: C4-C5 right paramedian spinal cord compression by extruded disc material of hyperintense signal (yellow arrowheads). At the same level, the presence of a nonspace-occupying intramedullary lesion of hyperintense signal (red arrowheads). (**D**) isointense signal in T1W (yellow arrowheads) in the absence of enhancement in the contrastographic phase, compatible with nonspecific spinal cord damage (edema, gliosis).

There was evidence of a nonspace-occupying intramedullary lesion at the level of C4-C5 of hyperintense signal in T2-weighted and isointense signal in T1-weighted in the absence of enhancement in contrastographic phase, compatible with nonspecific spinal cord damage (Figure 3).

In accordance with the owner, a conservative treatment with meloxicam (initial dose at 0.2 mg/kg on the first day, then maintenance dose at 0.1 mg/kg, once daily for 5 days; Metacam, Boehringer Ingelheim Animal Health Italia S.p.A, Italy) and exercise restriction for 4 weeks was prescribed. During the treatment period, the dog underwent several physiotherapy sessions. The physiotherapy protocol consisted of kneading massage (pétrissage technique), laser therapy of the cervical region, and passive range of motion (PROM) with the patient positioned on a mattress, first on one side and then on the other. When the dog acquired limb movements, the underwater treadmill was added at a minimum speed of 2 km/h, with the water level at the greater trochanter, gradually increasing the activity time from 5 to 15 min. Finally, when the patient began to take a few steps, the use of a circular proprioceptive platform and the inclusion of obstacle courses were incorporated into the protocol. The patient showed progressive improvement until complete resolution of the initial neurological symptoms at the 4-month follow-up. At 7 months after the initial presentation, a telephone follow-up with the owner confirmed the absence of symptoms.

## 3. Discussion

The two most common types of extrusion of non-degenerative or minimally degenerative nucleus pulposus are acute non-compressive nucleus pulposus extrusion (ANNPE) and hydrated nucleus pulposus extrusion (HNPE) [13]. As in other neurological conditions, magnetic resonance imaging is considered the gold standard imaging modality for HNPE diagnosis [14,16]. It is characterized by a narrowed intervertebral disc space with a decrease in signal intensity on T2W sequences [14], ventral extradural compressive material, which is homogenously hyperintense on T2W sequences and hypointense on T1W sequences, lying immediately dorsal to the affected intervertebral disc [13]. HNPE can have a characteristic bilobed or “seagull appearance” in transverse sequences. This typical shape is possibly explained by the location of the compressive material just ventral to the intact meningovertebral ligament [15]; a T2W hyperintense intramedullary lesion could be present in the overlying spinal cord (spinal cord damage or edema), and extruded nucleus pulposus can demonstrate a degree of contrast enhancement [8,14]. In our case, the diagnostic features in the MRI of both HNPEs reflect the current literature. Although MRI is diagnostic of choice for the diagnosis of HNPE, CT-angiography shows HNPE as a hypodense, well-demarcated extradural compressive lesion with an increased border immediately dorsal to the intervertebral disc space with a sensitivity of 91% and a specificity of 100% to differentiate cervical HNPE from IVDE [6]. HNPE has been well described in past years’ studies; signalment and clinical presentation in this case report were similar to those reported in the literature [5,8,9,10,14]. Treatment can be medical or surgical, and currently, it is uncertain which one represents the best option. No significant differences in short- and long-term outcomes were found in two studies between conservative and surgically treated groups, regardless of the neurological presentation [10,14]. The outcome seems to depend on the severity of clinical signs, with unsuccessful cases demonstrating tetraplegia with respiratory compromise at initial presentation [14]. To date, surgical treatment is chosen when there is evidence of severe spinal cord compression associated with severe neurological signs and respiratory compromise [3,14]. Surgical treatment typically consists of decompressive surgery using a ventral slot procedure, while medical management consists of limited exercise in combination with appropriate nursing care, physiotherapy, and, eventually, anti-inflammatory and analgesic medications [6,17]. Despite the severity of the symptoms, our patient achieved complete recovery with conservative treatment. This result was in accordance with previous studies suggesting an optimal recovery in patients with cervical compressive HNPE [6,8,10,13,18]. In our case, compression at the two herniation sites showed a moderate degree of spinal cord compression, and the dog showed non-ambulatory tetraparesis in the absence of respiratory distress. The rapid improvements that can be seen in clinical signs reported after the initiation of medical treatment may suggest that spinal cord contusion plays an important role in the pathophysiology of HNPE, overshadowing surgical decompression in these cases [3]. In fact, some studies have shown spontaneous regression of extradural disc material in animals under medical management that underwent a control MRI [3,18]. In contrast to Hansen type I intervertebral disc herniations (IVDHs), where the extruded material is calcified and hard [19,20], in HNPE, the extruded material has a gelatinous to liquid consistency [8]. Therefore, in HNPE, it is possible for the nucleus pulposus extruded into the spinal canal to spontaneously reabsorb in a relatively short period of time, subsequently resolving the spinal cord compression [18]. As in a previous study, the MRI appearance of HNPEs and the dog’s symptomatology influenced the therapeutic decision, as it was seen that HNPEs with heterogenic appearance were more likely to be treated surgically, assuming the presence of at least partly solid and less liquid disc material causing long-term compression without surgery [7]. In fact, a recent study demonstrated 2 cases that had multiple sites of cervical spinal cord compression caused by IVDH Hansen type I treated with a double adjacent ventral slot [21]. In the present study, the dog was treated with conservative therapy and rested for an initial period. Subsequently, individualized physiotherapy (hydrotherapy) was performed. Despite the presence of double adjacent cervical HNPEs, the patient achieved complete resolution of neurological symptoms after 4 weeks, consistent with data in today’s literature. Multiple-site IVDHs have been described in dogs and reported to be caused by the extrusion of degenerate nucleus pulposus [20,21,22].

## 4. Conclusions

To the authors’ knowledge, this is the first case report of multiple adjacent cervical HNPEs in a dog. This finding is an important addition to the current literature and confirms the possibility of multiple lesions in HNPE as well as in other IVDH. In our case, the outcome was similar to that of dogs with single HNPE treated conservatively. This suggests that therapy and outcome may not differ between single- and multiple-site HNPE. Further studies with larger numbers of cases are needed to investigate the clinical diagnostic aspects and to describe the treatment and outcome of dogs with multiple-site HNPE.

## Data Availability

The data presented in this study are available on justified request from the corresponding author.

## References

[1] De Decker S., Fenn J. (2018). Acute Herniation of Nondegenerate Nucleus Pulposus: Acute Noncompressive Nucleus Pulposus Extrusion and Compressive Hydrated Nucleus Pulposus Extrusion. Vet. Clin. N. Am. Small Anim. Pract..

[2] Fenn J., Olby N.J., Canine Spinal Cord Injury Consortium (CANSORT-SCI) (2020). Classification of Intervertebral Disc Disease. Front. Vet. Sci..

[3] Manunta M.L., Evangelisti M.A., Bergknut N., Grinwis G.C., Ballocco I., Meij B.P. (2015). Hydrated nucleus pulposus herniation in seven dogs. Vet. J..

[4] Kristiansen K.V., Schmökel H., Vermeire S. (2022). Hydrated Nucleus Pulposus Extrusion in Dogs: Thoracolumbar Compared to Cervical Cases. Vet. Comp. Orthop. Traumatol..

[5] Falzone C. (2017). Canine acute cervical myelopathy: Hydrated nucleus pulposus extrusion or intraspinal discal cysts?. Vet. Surg..

[6] Royaux E., Martlé V., Kromhout K., Van der Vekens E., Broeckx B.J., Van Ham L., Gielen I. (2016). Detection of compressive hydrated nucleus pulposus extrusion in dogs with multislice computed tomography. Vet. J..

[7] Nessler J., Flieshardt C., Tünsmeyer J., Dening R., Tipold A. (2018). Comparison of surgical and conservative treatment of hydrated nucleus pulposus extrusion in dogs. J. Vet. Intern. Med..

[8] Dolera M., Malfassi L., Marcarini S., Mazza G., Sala M., Carrara N., Facchini R.V., Finesso S. (2015). Hydrated nucleus pulposus extrusion in dogs: Correlation of magnetic resonance imaging and microsurgical findings. Acta Vet. Scand..

[9] Hamilton T., Glass E., Drobatz K., Agnello K.A. (2014). Severity of spinal cord dysfunction and pain associated with hydrated nucleus pulposus extrusion in dogs. Vet. Comp. Orthop. Traumatol..

[10] Borlace T., Gutierrez-Quintana R., Taylor-Brown F.E., De Decker S. (2017). Comparison of medical and surgical treatment for acute cervical compressive hydrated nucleus pulposus extrusion in dogs. Vet. Rec..

[11] Farré Mariné A., López Beltran M., Ortiz Nisa S., Luján Feliu-Pascual A. (2024). Myelo-CT imaging findings in 15 dogs with surgically-treated cervical acute compressive hydrated nucleus pulposus extrusion. Vet. Radiol. Ultrasound..

[12] Konar M., Lang J., Flühmann G., Forterre F. (2008). Ventral intraspinal cysts associated with the intervertebral disc: Magnetic resonance imaging observations in seven dogs. Vet. Surg..

[13] da Costa R.C., De Decker S., Lewis M.J., Volk H., Canine Spinal Cord Injury Consortium (CANSORT-SCI) (2020). Diagnostic Imaging in Intervertebral Disc Disease. Front. Vet. Sci..

[14] Beltran E., Dennis R., Doyle V., de Stefani A., Holloway A., de Risio L. (2012). Clinical and magnetic resonance imaging features of canine compressive cervical myelopathy with suspected hydrated nucleus pulposus extrusion. J. Small Anim. Pract..

[15] Kent M., Glass E.N., Song R.B., Warren J.D., de Lahunta A. (2019). Anatomic description and clinical relevance of the meningovertebral ligament in dogs. J. Am. Vet. Med. Assoc..

[16] Tabbì M., Barillaro G., Interlandi C.D., Di Pietro S., Fugazzotto D., Costa G.L., Iannelli N.M., Macrì D., Ferrantelli V., Macrì F. (2023). Treatment of Canine Disc-Associated Cervical Spondylomyelopathy with a Cervical Distraction–Stabilization Technique (C-LOX Combined with LCP Plate) and Clinical Outcomes. Animals.

[17] Lowrie M.L., Platt S.R., Garosi L.S. (2014). Extramedullary spinal cysts in dogs. Vet. Surg..

[18] Kamishina H., Ogawa H., Katayama M., Yasuda J., Sato R., Tohyama K. (2010). Spontaneous regression of a cervical intraspinal cyst in a dog. J. Vet. Med. Sci..

[19] Brisson B.A. (2010). Intervertebral disc disease in dogs. Vet. Clin. N. Am. Small Anim. Pract..

[20] Cojocaru R.G., Sicoe B., Gaspar C., Grigoreanu A., Orghici G., Tibru I., Lacatus R. (2024). Case report: Double adjacent ventral slot in two medium-sized breed dogs. Front. Vet. Sci..

[21] Chang Y.P., Huang W.H., Lua W.Z., Wong W., Liu I.H., Liu C.H. (2023). Outcomes in Dogs with Multiple Sites of Cervical Intervertebral Disc Disease Treated with Single Ventral Slot Decompression. Vet. Sci..

[22] Guo S., Lu D., Pfeiffer S., Pfeiffer D.U. (2020). Non-ambulatory dogs with cervical intervertebral disc herniation: Single versus multiple ventral slot decompression. Aust. Vet. J..

